# Fluency-related Temporal Features and Syllable Prominence as Prosodic Proficiency Predictors for Learners of English with Different Language Backgrounds

**DOI:** 10.1177/00238309211040175

**Published:** 2021-09-03

**Authors:** Heini Kallio, Antti Suni, Juraj Šimko

**Affiliations:** University of Helsinki, Finland

**Keywords:** Spoken second or foreign language assessment, native language transfer effect, temporal fluency, prominence

## Abstract

Prosodic features are important in achieving intelligibility, comprehensibility, and fluency in a second or foreign language (L2). However, research on the assessment of prosody as part of oral proficiency remains scarce. Moreover, the acoustic analysis of L2 prosody has often focused on fluency-related temporal measures, neglecting language-dependent stress features that can be quantified in terms of syllable prominence. Introducing the evaluation of prominence-related measures can be of use in developing both teaching and assessment of L2 speaking skills. In this study we compare temporal measures and syllable prominence estimates as predictors of prosodic proficiency in non-native speakers of English with respect to the speaker’s native language (L1).

The predictive power of temporal and prominence measures was evaluated for utterance-sized samples produced by language learners from four different L1 backgrounds: Czech, Slovak, Polish, and Hungarian. Firstly, the speech samples were assessed using the revised Common European Framework of Reference scale for prosodic features. The assessed speech samples were then analyzed to derive articulation rate and three fluency measures. Syllable-level prominence was estimated by a continuous wavelet transform analysis using combinations of F0, energy, and syllable duration.

The results show that the temporal measures serve as reliable predictors of prosodic proficiency in the L2, with prominence measures providing a small but significant improvement to prosodic proficiency predictions. The predictive power of the individual measures varies both quantitatively and qualitatively depending on the L1 of the speaker. We conclude that the possible effects of the speaker’s L1 on the production of L2 prosody in terms of temporal features as well as syllable prominence deserve more attention in applied research and developing teaching and assessment methods for spoken L2.

## 1 Introduction

The ability to speak proficiently is often the ultimate goal associated with mastering a second or foreign language (L2). Oral L2 proficiency includes many facets, but a core skill in spoken communication is pronunciation on both segmental (individual sounds) and suprasegmental or *prosodic* (e.g., intonation, rhythm, and stress) levels. While the well-known language learning theories have focused on the segmental aspects of pronunciation (Best et al., 1994; Flege, 1995; Kuhl, 1993), prosody has received increased attention among L2 researchers in the past decade (Isaacs, 2018). Prosodic features form the backbone of speech, providing the structure that links individual sounds to one another, and shapes both linguistic and paralinguistic meanings. The investigation of prosodic factors that can affect spoken language proficiency is thus of interest not only to basic science but also to applied research related to language pedagogy and assessment. This study continues the scrutiny of prosodic aspects of oral language proficiency with a focus on global and local temporal features in speech.

Since the prosodic systems of languages differ substantially (Hirst & Di Cristo, 1998), language learners may experience a transfer effect from their native language (L1) to the target language. Previous research results indicate that compared to segmental errors, prosodic deficiencies often attributable to the transfer effect are even more detrimental in achieving intelligibility, comprehensibility, and fluency in the L2 (Anderson-Hsieh et al., 1992; Kang, 2012; Munro & Derwing, 1999; Pinget et al., 2014). Many issues, however, remain little studied. For example, most studies have focused on temporal measures that occur globally in speech as predictors of fluency or oral proficiency, neglecting stress, which has language-dependent and more local acoustic realizations (Cucchiarini et al., 2002; Derwing et al., 2004; Kormos & Dénes, 2004; Pinget et al., 2014). It has also been common to study the speech of language learners from only one L1 background at a time (Derwing et al., 2004; Kormos & Dénes, 2004; Trofimovich & Baker, 2006) or pool L2 speech data together regardless of the speaker’s L1 (Cucchiarini et al., 2002; Kang, 2012; Pinget et al., 2014). Comparative research, in turn, has usually included L2 learners from two typologically distant languages and observed the effect of the speaker’s L1 from the perspective of perceived foreign accent (Derwing et al., 2006; Huang et al., 2016; Winke & Gass, 2013). A few studies have included three or more L1s (Altmann, 2006; Anderson-Hsieh et al., 1992; Derwing & Munro, 1997; Kijak, 2009), but only Altmann (2006) and Kijak (2009) have compared L2 stress production with respect to the speaker’s L1. The comparison provided by the dissertations of Altmann (2006) and Kijak (2009) focused mainly on the effect of lexical stress position in the L1 on the placement of stress in the L2.

In this study, we investigate prosodic features as predictors of L2 English speaking proficiency from two perspectives: measuring temporal features related to fluency, and comparing syllable-level stress realizations of native and L2 speakers. The focus of the paper is on the comparison of the two methods in predicting assessments of prosodic proficiency. In addition, we examine the L2 speech of learners with four different L1 backgrounds: Czech, Slovak, Polish, and Hungarian. The current set of languages differs from that in previous research with respect to typological distance, which broadens the research scope of L1 effects on L2 speech to more fine-grained acoustic differences that remain invisible when studying typologically very distant languages. In the current study, we are interested in whether prominence as an acoustic realization of stress and a local aspect of prosody can predict L2 learners’ oral proficiency better than traditionally used temporal measures, and whether speakers’ L1s play a role in proficiency assessments in spite of the relative closeness of the L1s.

### 1.1 Prosodic measures of L2 proficiency

L2 prosody has generally been studied from the perspectives of foreign accent and/or fluency (Bosker et al., 2013; Cucchiarini et al., 2002; de Mareüil & Vieru-Dimulescu, 2006; Derwing et al., 2004; Kaglik & de Mareüil, 2010). Prosodic features have, however, also been found to explain a substantial amount of the variance in general oral proficiency ratings (Kang et al., 2010). In particular, temporal features related to speech fluency have proven to be strong predictors of prosodic competence (Cheng, 2011; Hönig et al., 2010; Kallio et al., 2017; Kang & Johnson, 2018), which is why we start by reviewing studies related to the temporal fluency of L2 speech. Although fluency research has commonly focused on measuring temporal features, arguments have been raised that fluency involves both temporal and intonational phenomena (Kang et al., 2010; Kormos & Dénes, 2004). Furthermore, it has been noted that prosodic errors tend to accumulate in L2 speech so that disfluencies affect stress production (Rasier & Hiligsmann, 2007). This means, for example, that unintentional pausing and hesitation in L2 speech can cause the speaker to stress wrong words or syllables. We suspect that the direction of this effect can also be reversed: difficulties in achieving temporal fluency in L2 speech might be based on incorrect production of word or sentence stress. Thus, introducing the evaluation of local prominence-related measures to complement the more traditionally used temporal features of L2 speech can help improve assessment methods and define the concept of language proficiency in terms of prosody. The following sections review the literature on temporal fluency in L2 speech and L2 stress production realized as syllable prominence.

#### 1.1.1 Temporal fluency

Fluency is one of the most commonly used terms in language pedagogy and testing and it is generally included in many assessment criteria of oral L2 skills, such as the Common European Framework of Reference (CEFR) for languages (Council of Europe, 2017), International English Language Testing System (IELTS; British Council, 2019), Pearson (Pearson, 2017), and ETS (Educational Testing Service, 2014). It is also very likely the primary measure that ordinary interlocutors assess in everyday interaction. However, there are several ways to approach fluency (Chambers, 1997; Huhta et al., 2019). Lennon (2000) presents two types of fluency definitions: a broad one and a narrow one. The broad sense corresponds to a higher-order, general proficiency, while the narrower definition refers to spoken performance and more precisely to the temporal properties and “smoothness” of the speech. The present study approaches fluency from the latter, narrower perspective, which could also be referred to as utterance fluency (Segalowitz, 2010). Tavakoli and Skehan (n.d.), in turn, distinguish three components related to utterance fluency: (1) *speed fluency*, referring to the speed at which speech is delivered (usually measured as speech rate, articulation rate, mean length of syllables, or mean length of runs); (2) *breakdown fluency*, referring to pausing phenomena (usually measured as the frequency or length of silent and filled pauses); and (3) *repair fluency*, referring to false starts, corrections, and repetitions (usually measured as frequency or length of the respective disfluencies).

Many phonetic studies have used measures related to these components, and measures of speed and breakdown fluency in particular have been found to correlate with fluency assessments. The findings provide, however, a somewhat inconsistent picture: Derwing et al. (2004) investigated the speech of Mandarin-speaking learners of English and found significant correlations between L2 fluency ratings and *pausing* as well as *articulation rate*. Kormos and Dénes (2004), in turn, studied the link between utterance fluency measures and perceived fluency of Hungarian L2 speech. They found the *speech rate, mean length of utterance, phonation–time ratio*, and *number of stressed words produced per minute* to be the best predictors of fluency ratings. Cucchiarini et al. (2002) compared the acoustic fluency correlates of read and spontaneous speech and found that *speech rate* was the best predictor of fluency scores in both speech types, especially for the beginner level, while *articulation rate* had a strong correlation with perceived fluency in read speech but not in spontaneous speech. Bosker et al. (2013) studied the link between utterance fluency measures and perceived fluency in spontaneous speech with L2 Dutch and found that *filled and silent pauses* and *the mean length of pause* together with the *mean length of syllable* are significant predictors of fluency ratings. Préfontaine et al. (2016) investigated how temporal measures predicted untrained listeners’ ratings of fluency in French as a second language. They found that *the mean length of runs* (a combination of speed and breakdown features) and *articulation rate* were better predictors of perceived fluency than pause measures.

The diversity of these results can be attributed to different conceptualizations and operationalizations of fluency and its measures as well as to the different types of analyzed data. There are, for example, some inconsistencies in defining pauses: depending on the study and speech material, the pause threshold ranges from 50 ms to as long as 3 s (Bosker et al., 2013; Derwing et al., 2004; Kallio et al., 2017; Kormos & Dénes, 2004; Préfontaine et al., 2016), which can result in characterizing similar data with different parameter values. Some acoustic measures, in turn, are confounds: for example, both speech rate and mean duration of pause are related to the duration of pauses in the speech signal and are therefore interrelated, which can affect the interpretation of the results. The present study pays attention to the correlation of temporal measures. Another reason for the conflicting results might arise from the proficiency levels of speakers: for example, Cucchiarini et al. (2002) found that the predictive power of different acoustic measures varied not only between speech styles, but also between beginner and intermediate level speakers.

Some disfluencies occur differently depending on the context. Filled pauses are generally more typical for spontaneous speech than read speech, but the use of pauses can also be a language-specific phenomenon; for example, Campione and Véronis (2002) noted that Italians generally make shorter pauses than French, English, and German speakers, whereas Spanish speakers tend to pause longer than the other language groups. In French conversational speech filled pauses seem to be frequent, while silent pauses are used parsimoniously and spared for syntactic structuring (Duez, 1982). Finnish speakers, in turn, tend to use even longer silent pauses (while reading) than non-native speakers of Finnish (Toivola et al., 2009). These findings encourage further research to take into account the possible L1-specific differences in the use of pauses and other fluency-related phenomena.

#### 1.1.2 Syllable prominence

In stress languages, one syllable in a word is usually pronounced with more *prominence* to make it stand out acoustically and perceptually. Languages differ in both the positioning and acoustic realization of stress (Ladefoged & Johnson, 2014), and the correct production of language-specific stress patterns is important in both intelligibility and fluency of L2 speech (Field, 2005; Hahn, 2004; Kormos & Dénes, 2004; Munro, 1995; Trofimovich & Baker, 2006; Wennerstrom, 2000). Acoustically, a stress-bearing syllable is typically characterized by an increase in F0, duration, and intensity (Cruttenden, 1997; Fant & Kruckenberg, 1994; Lehiste, 1969; Lieberman, 1967; Vainio & Järvikivi, 2006). These parallel signal characteristics combine in a complex manner, which makes prominence difficult to quantify.

Previous research on L2 stress production as well as automatic assessment of L2 prosody has focused either on the placement of word and/or sentence stress or the frequency of stressed words/syllables in speech (Altmann, 2006; Guion, 2005; Kang & Johnson, 2018; Kijak, 2009; Kormos & Dénes, 2004; Wennerstrom, 2000; Zechner et al., 2009). Automatic stress detection systems developed for L2 learning purposes usually use binary or ternary classification of stress (stressed/unstressed or primary stress/secondary stress/unstressed) based on syllable-level F0, duration, and intensity as well as complementary features, such as root mean square (RMS) energy range and F0 slope, spectral tilt, or relative sonority levels (Ferrer et al., 2015; Li et al., 2018; Tepperman & Narayanan, 2005; Yarra et al., 2017). They focus on identifying word stress and are suitable for evaluating the placement and frequency of stressed syllables, but differences in the use of acoustic features for stress realizations remain ignored.

In this study, we estimate syllable prominence using a recent methodology based on the continuous wavelet transform (CWT) of prosodic features. The method has been shown to provide strong correlations with perceptual prominence (Eriksson et al., 2018; Suni et al., 2017) as well as promising results in predicting prosodic proficiency of L2 learners (Kallio et al., 2018, 2020). Compared to previous methods used in detecting syllable stress, we use relative prominence values for all syllables in an utterance instead of categorizing syllables as stressed or unstressed. This allows us to take into account both word and sentence level production of prominence and compare the stress realizations between speakers in more detail. In Kallio et al. (2018, 2020), we compared the estimated syllable prominence values between L2 and native speakers of Finland Swedish from read utterances and found that the higher the correlation in prominence values between native and L2 speakers, the better the prosodic proficiency grades of L2 speakers. However, the previous studies provided somewhat unexpected results regarding the feature combinations that best predicted the proficiency scores: energy and duration together provided the best results, which indicate that in the speech of L2 learners there might be a revealing pattern in the use of these features when producing prominence. The differences between the target language and speakers’ L1 may affect the use of F0, duration, and intensity in producing prominence, and these differences should be taken into account when syllable prominence is used as a measure of L2 proficiency.

### 1.2 Comparison of languages in this study

This study investigates the temporal features and syllable prominence of L2 English produced by speakers with either Czech, Slovak, Hungarian, or Polish as a L1. Czech, Slovak, and Polish are West-Slavic Indo-European languages, while Hungarian belongs to the Finno-Ugric branch of the Uralic language family. However, Czech, Slovak, and Hungarian share a number of prosodic characteristics and other linguistic features, which have been hypothesized to stem from historical convergence as well as direct linguistic influence (Newerkla, 2000). The speech data used in this study was originally collected in collaboration by research affiliations in the four countries aiming to study the similarities and differences in the L2 English accents of Czech, Slovak, Hungarian, and Polish speakers. This collaboration is the main motivation for choosing these languages in the current research, but the four languages also provide an interesting combination of typological similarities and differences, in particular in terms of prosody and realization of lexical stress. In the following sections we compare lexical and phrasal stress as well as acoustic realizations of prominence of the chosen L1s and the target language, English.

#### 1.2.1 Lexical stress

The difficulties that the English stress system has posed to language learners have been noted in various sources (Hahn, 2004; Halle & Keyser, 1971; Kormos & Dénes, 2004; Mixdorff & Ingram, 2009; Trofimovich & Baker, 2006; Wennerstrom, 2000). Regarding the current study, the most relevant differences between the target language English and the learners’ L1s are the positioning and acoustic realization of lexical stress. Czech, Slovak, Hungarian, and Polish all have fixed word stress, as opposed to English, which has a far more complex stress system (Halle & Keyser, 1971). In Czech, Slovak, and Hungarian, lexical stress falls on the first syllable of the word, and in Polish on the penultimate one. Lexical stress in English, in turn, is variable, making it less predictable than languages with fixed stress (Bolinger, 1965; Halle & Keyser, 1971).

The primary prosodic feature signaling prominence in English is said to be duration: stressed syllables in English are considerably longer than unstressed syllables, and F0 and intensity remain contributing markers of stress (Ladefoged & Johnson, 2014). Hungarian, Czech, and Slovak have a phonemic quantity distinction for vowels (Hungarian also has such a distinction for consonants), which may weaken the role of duration as a signal for prominence in these languages: it has been claimed that if a language uses an acoustic property for one function, it will not use the same property for another function (Remijsen, 2002). Based on this Functional Load Hypothesis (FLH), Hungarian, Slovak, and Czech would prefer other acoustic cues instead of duration when marking prominence. The applicability of the FLH, however, has been evaluated with results from a database of 140 languages (Lunden et al., 2017). While most languages seem to fail to follow the FLH, available studies reported in the Stress Correlate Database (Lunden & Kalivoda, 2021) support the tendency in Czech and Hungarian.

While in Hungarian F0 patterns have been assumed to be determined primarily by sentence information structure (Varga, 2002), Vogel et al. (2015) have indeed found F0 to also be the strongest indicator of Hungarian lexical stress and suggest that duration is avoided as a cue to prominence. Studies on acoustic correlates of stress in Czech, in turn, indicate that the realization of prominence in the language is not straightforward: Dubeda and Votrubec (2005) found F0 as the strongest and duration as the weakest predictor of stress, while Skarnitzl and Eriksson (2017) proved both features meaningful but found them to behave in a counter-intuitive way, resulting in lower F0 and shorter duration in stressed syllables. The results of Skarnitzl and Eriksson (2017) indicate that the acoustic characteristics of prominence might be delayed to the following syllable in Czech. The acoustics of Slovak stress has been studied only marginally, but the absence of clear prosodic marking of prominence is also noted in Slovak (Benuš et al., 2014). However, duration has been found to have very little effect on either the production or the perception of prominence (Benuš & Mády, 2010, 2012). Polish, as opposed to the other L1s in this study, does not have a quantity distinction in its vowels (Dogil & Williams, 1999). Malisz and Wagner (2012), however, found that F0 and intensity serve as main determinants of overall prominence in Polish, leaving duration less significant. Their results support the previously found importance of F0 in the Polish stress system (Jassem, 1962).

Relatively few studies have been done on the production of English stress patterns by speakers of Czech, Slovak, Hungarian, and Polish (Archibald, 1993; Bakti & Bóna, 2014; Bilá & Zimmermann, 1999; Weingartová et al., 2014). For Hungarian learners of English, studies have focused only on stress placement, explaining production errors with both fixed word stress and quantity-sensitivity (Archibald, 1993; Bakti & Bóna, 2014). Archibald (1993) also found some evidence of L1 stress transfer in the speech of Polish learners of English, but a comprehensive study on the production of English stress by Hungarian and Polish speakers is still lacking. For Slovak and Czech learners of English, the use of duration in marking prominence has been under inspection (Bilá & Zimmermann, 1999; Weingartová et al., 2014). Bilá and Zimmermann (1999) found evidence that Slovak speakers face difficulties in using duration to differentiate stressed and unstressed syllables in English. Weingartová et al. (2014), in turn, investigated prominence patterns in Czech-accented English and found the duration ratio between stressed and unstressed syllables to be the most significant correlate of language learners’ proficiency.

In English, the contrast between stressed and unstressed syllables is manifested relatively strongly, often resulting in reduction of the unstressed vowels to a schwa (Bolinger, 1965; Halle & Keyser, 1971). In Czech, Slovak, Hungarian, and Polish, vowel reduction does not operate as a stress correlate (Dancovicova & Dellwo, 2007; Jassem, 1962), but the languages are not entirely immune to reduction processes: a centralization effect has been found at least in Polish (Rojczyk, 2019) and Slovak (Benuš & Mády, 2010). In all four languages, however, stress contrasts seem to be weaker compared to English. This might be due to the fact that, in contrast to English, the fixed word stress is not important for lexical contrast in Czech, Slovak, Hungarian, or Polish. Acoustic marking of word stress is found to be absent in Czech (Skarnitzl & Eriksson, 2017), realization of prominence in Hungarian has been deemed to be relatively weak (Vogel et al., 2015), and Mocova (2012) claims that “Slovak stress is one of the weakest among European languages.” Word stress in Polish, in turn, has been characterized as “at best weakly realized” (Dogil & Williams, 1999) and acoustic marking of prominence has been found at the phrase-level only (Cwiek & Wagner, 2018).

#### 1.2.2 Sentence stress

In addition to lexical stress, prominence patterns also arise from temporally wider prosodic phenomena, such as sentence stress. In English, the most prominent part of a phrase (without a specific focus condition) is usually in the final position, and elements before sentence stress are prosodically reduced (Dubeda & Mády, 2010; Roach, 2000). However, English word order is relatively fixed, and the use of, for example, focus (broad vs. narrow) can be confirmed with the placement of sentence stress (Dubeda & Mády, 2010). Similar findings on the position of sentence stress have been presented for Polish and Czech, but in contrast to English, there is a tendency to keep the prominent part at the end of a phrase regardless of the focus condition, which is permitted by relatively free word order in these languages (Dubeda & Mády, 2010; Eschenberg, n.d.). Hungarian, also a language with free word order, differs from Polish and Czech with regards to sentence stress: Hungarian has a stronger prominence in the beginning of a phrase as a default (Dubeda & Mády, 2010; Varga, 2002).

The acoustic analysis of sentence stress often focuses on finding the F0 peaks in a phrase, but the contributions of intensity and duration have also received attention. In a study by Szalontai et al. (2016) about Hungarian word and sentence stress, syllable duration was significantly affected by both word-level and phrasal stress patterns, but F0 and intensity contributed only to phrasal stress. Cwiek and Wagner (2018) studied Polish word and sentence stress and found duration as the most significant marker of prominence when word and sentence stress were realized in the same syllable. For sentence stress alone, however, F0, intensity, and spectral balance were more salient markers than duration. Similarly, Igras and Ziolko (2016) found F0 and energy parameters as best measures of Polish sentence stress, while syllable duration increased only slightly. For the Czech sentence stress, F0 seems to be the primary marker and duration the least significant (Dubeda and Votrubec, 2005). Realization of sentence stress in Slovak has been less studied, but Benuš et al. (2014) modeled accentual phrase intonation in Slovak and Hungarian and concluded that F0 contour patterns have a falling tendency in Hungarian, while the Slovak F0 contours rise before they fall.

To conclude, the fixed word order in English permits the placement of sentence stress to vary more than in other languages in this study, which may inhibit the functional use of sentence stress in non-native speakers of English. However, language-specific characteristics in the realization of sentence stress can also result in differing manifestations regarding both prominence and overall temporal features in L2 speech.

### 1.3 Research questions

The main objective of this study is to compare fluency-related temporal measures and CWT-based continuous syllable prominence estimates as predictors of L2 prosodic proficiency with respect to a speaker’s L1. The research questions that the current study aims to address are as follows.

(RQ1) Can temporal measures be used to predict prosodic proficiency assessments of L2 speakers from different L1 backgrounds?
(RQ2) Can syllable-level prominence realizations be used in predicting the same prosodic proficiency assessments?
(RQ3) How do the two methods complement each other?

To answer RQ1, we study the effect of various temporal measures and their interactions with speakers’ L1s on prosodic proficiency assessments using statistical models designed for ordered categorical data. In order to address RQ2, we calculate correlations between syllable-level prominence estimates for sentences produced by L1 and L2 speakers, and evaluate the potential influence of the speaker’s L1 on the relationship between these prominence-based measures and assessment grades. To answer RQ3, we compare statistical models with temporal measures and CWT-based syllable prominence estimates as predictors of prosodic proficiency.

We expect both methods to be applicable for distinguishing speakers with different proficiency levels, following the findings of Kallio et al. (2017, 2020). Based on the previous studies discussed above, we expect the temporal measures to be good predictors of prosodic proficiency regardless of the speaker’s L1. However, we acknowledge that some differences in these measures may arise from the possible language-specific tendencies in, for example, the use of pauses.

With regard to the CWT-based method, we expect the salience of different prosodic characteristics (F0, duration, and energy) to be language-dependent in prominence production and, therefore, the local prominence measures to reveal differences arising from language-specific acoustic realizations of lexical and phrasal stress. Based on our previous findings (Kallio et al., 2020), the best feature combination for predicting prosodic proficiency in L2 also depends on the strongest indicator of stress in the target language. In English, duration is considered to be that indicator, which supports the presumption that duration would be included in the best feature combinations for capturing syllable prominence characteristics correlating with proficiency assessments.

## 2 Methods

### 2.1 Participants

The speech data used in this study was provided by four universities: Constantine the Philosopher University in Nitra, Slovakia; Metropolitan University Prague, Czech Republic; University of Łódz, Poland; and the Hungarian Academy of Sciences. Adult learners of English with Slovak (*N* = 14), Czech (*N* = 14), Polish (*N* = 17), or Hungarian (*N* = 16) as their L1 were previously recorded in these universities. Each university aimed at recruiting participants with differing levels of English skills, but the overall proficiency levels of the L2 speakers were not systematically controlled for prior to recording of the material.

Speech samples from 14 participants from each L1 group were used in this study. Thus, all participants from the Slovak and Czech L1 groups were included in the current study. Two speakers from the Hungarian data were excluded due to poor audio signal quality, leaving 14 speakers in this group also. In order to have an equal number of speakers per L1 group, three speakers from the Polish data were subsequently excluded by the authors: one perceived as a low level (A1 or lower) speaker, one as an intermediate level (B1), and one as a proficient speaker (C1/C2). Since the proficiency levels were not originally balanced between the L1 groups, the authors did not want to interfere with the proficiency distributions of the groups. This method was also considered less likely to cause bias than excluding samples randomly due to the relatively small number of speakers in the group (in an unfortunate case, all three random samples could have represented the same proficiency level). The proficiency levels used here are based on the CEFR for languages (North, 2007), which is a guideline for describing the achievements of L2 learners. It provides six reference levels (A1, A2, B1, B2, C1, and C2, from the lowest to the highest) that are commonly used as the European standard for grading an individual’s language proficiency.

In order to compare the prominence realizations between L1 and L2 speakers, data from seven native speakers of English were recorded in a soundproof studio in Constantine the Philosopher University in Nitra reading the same narrative as previously recorded L2 speakers. Five (male) speakers were native speakers of British English and two (one male and one female) were native speakers of American English.

### 2.2 Recordings

The recordings were done one participant at a time using either a recording studio or some other quiet space with a portable recording device. All participants were given at least 2 min to get acquainted with the text and the opportunity to ask if they did not understand some words. Pronunciation instructions, however, were not given. The participants were then asked to read the text aloud as if they were telling the story to someone. If they stumbled, they could start again from the beginning of a sentence.

The narrative text consisted of 16 sentences and the full recordings were approximately 1.5 min long. A subset of four utterances was selected for this study using the following principle: the target utterances differ from each other with respect to utterance and word length, vocabulary, and structure. The same four utterances were extracted from the seven native speakers and 56 L2 speakers, resulting in 28 L1 speech samples and 224 L2 samples. The utterances were extracted as grammatical sentences and are listed in Table 1.

**Table 1.** Target utterances.

They said it was a five star hotel, but I wouldn’t give it one star.
There were big ships travelling past, and the sea was all polluted and brown, it looked horrible.
It was a sort of greeny black colour, and as we looked more closely, we realised that it was
full of frogs.
Instead we were very surprised to see lots of different types of vegetables: carrots, peas,
cabbage, and a big bowl of lettuce.

### 2.3 Human assessments

Assessments were performed by university students (Constantine the Philosopher University in Nitra, Metropolitan University Prague, University of Łódz, and Eötvös Loránd University in Budapest) majoring either in English or Phonetics. The assessors’ L1s were Czech (*N* = 7), Slovak (*N* = 7), Hungarian (*N* = 13), and Polish (*N* = 13). Since our assessors were non-native speakers of English, they were asked to provide a self-evaluation of their English skills based on the CEFR descriptors (North, 2007). The self-evaluated proficiency levels varied from B2 (advanced independent user) to C2 (advanced proficient user), but most assessors estimated themselves as C1-level (proficient) English users. When the assessment task was not a part of their studies, assessors were given a small reward.

Previous research suggests that both trained and untrained as well as native and non-native raters can assess L2 speech relatively consistently, but phonetic and/or linguistic training, experience, and specific rating instructions increase the inter-rater reliability (Brennan & Brennan, 1981; Cucchiarini et al., 2002; de Wet et al., 2009; Derwing et al., 2004; Huang et al., 2016; Kormos & Dénes, 2004; Munro, 2008; Rossiter, 2009; Thompson, 1991). In our study, all assessors had studied phonetics and/or English at a university level. In addition, the assessors were given an identical introductory lecture on the assessment of spoken language skills, including assessment exercises focusing on prosodic features. The assessors were also provided written instructions for the assessment task, which they subsequently conducted independently.

The L2 speech samples were first divided into two pseudo-randomized sets that included an equal number of samples from each L1 speaker group. Each assessor was given one of the two sets so that each speech sample was assessed by 19 or 20 assessors. The order of the speech samples within the sets were further randomized for each assessor to avoid possible bias from comparing consecutive samples. Native English samples were not assessed.

Note that each utterance was graded by assessors with several different L1s. Scrutinizing the possible effect of an assessor’s L1 is beyond the scope of this paper; however, the consistency of the assessments is tested with Cronbach’s alpha.

For grading the samples, a six-level proficiency scale for phonological control from the updated CEFR descriptors (Council of Europe, 2017) was used. Here we focus on the assessments of prosodic features, which is a subsection of the CEFR scale for phonological control and pays attention to features such as word-level and phrasal stress, rhythm, and intonation with respect to the perceived intelligibility of speech.

### 2.4 Temporal fluency measures

The speech samples used in this study were prepared for analysis using Praat (Boersma & Weenink, 2016). Utterance-sized samples were annotated at a syllable level (following the sonority sequencing principle, see e.g., Parker, 2011) using WebMAUS (Kisler et al., 2017), and annotations were checked manually. Disfluencies longer than 50 ms were marked for manual analysis. This cut-off point for pauses and other disfluencies is notably lower than in previous studies (Cucchiarini et al., 2010; De Jong et al., 2015; Derwing et al., 2004; Kormos & Dénes, 2004; Préfontaine et al., 2016) and is based on findings that many pauses shorter than 250 ms cannot be attributed to articulation (Campione & Véronis, 2002; Hieke et al., 1983) and auditory observations of the current speech data, where hesitation breaks as short as 50 ms were perceived. The variables were selected based on previous research on temporal fluency in a L2 (Bosker et al., 2013; Cucchiarini et al., 2010; Derwing et al., 2004; Kormos & Dénes, 2004; Préfontaine et al., 2016).

The acoustic variables measured were articulation rate (ARTRATE), silent pause–time ratio (SP-RATIO), silent pause frequency (SP-FREQ), filled pause–time ratio (FP-RATIO), filled pause frequency (FP-FREQ), correction/repetition–time ratio (CR-RATIO), and correction/repetition frequency (CR-FREQ), following the study by Kallio et al. (2017). Articulation rate was calculated as syllables produced per second excluding all disfluencies and pauses longer than 50 ms. Disfluency frequencies are measured as the number of each type of disfluency per utterance. Disfluency–time ratios, in turn, were derived by dividing the total duration of each disfluency per utterance with the total duration of the utterance. The silent pause–time ratio is an equivalent of the more commonly used phonation–time ratio, but it is easier to compare to the other selected variables, because they are considered as types of disfluencies. These measures are hereafter referred to as “temporal fluency measures” or “temporal measures.”

### 2.5 CWT-based prominence estimation

The prominence estimates for individual syllables were obtained using a continuous wavelet analysis technique originally developed for word prominence detection and described by Suni et al. (2017). The Wavelet Prosody Toolkit used in this study is available online (Suni, 2019).

CWT is a technique that decomposes a signal to a range of frequency components using a convolution of the signal with a scaled wavelet function. The wavelet function is scaled (stretched in time and amplitude) to obtain the components of appropriate frequencies. Applying the CWT decomposition to prosodic signals, as used here, yields the components roughly corresponding to prosodic events at various time scales, such as syllable, word, or phrase levels of prosodic analysis.

For the utterance-sized samples used in study, annotated at a syllable level (yielding syllable durations), F0 contours were extracted using sample-dependent settings to avoid measurement errors arising from speaker- and sample-based F0 characteristics. Syllables with identical labels were numbered in order to separate them in the correlation analysis phase. Energy envelopes were acquired by band-pass filtering the speech signals between 400 and 5000 Hz and then extracting a smoothed Hilbert envelope.

The extracted F0 and energy envelope signals were sampled at 200 Hz and pre-processed using the methods described in detail by Suni et al. (2017). In addition, the duration signal was constructed in the following way: the value of each syllable duration was placed at the mid-time point of the unit, and a measure of difference between durations of adjacent syllables was placed on the boundary of respective syllables, and then the points were connected using cubic interpolation to form a smooth duration signal.

In order to evaluate which signal attributes correspond best to assessments of prosodic proficiency, we compared the CWT-based prominence estimates obtained from seven combinations of fundamental frequency (F0), energy envelope (en), and duration (dur): with each signal separately, all three pairs of signals, and the combination of all three signals.

**Figure 1.**
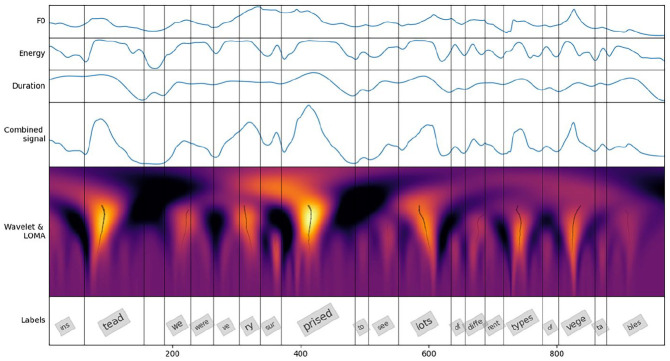
Continuous wavelet transform (CWT) prominence estimation method. Three upper panes show the prosodic signals (F0, en, and dur in the text), and the fourth pane shows the combined signal. The fifth pane depicts the CWT scalogram of the combined signal with the light color representing peaks; the lines of maximum amplitude (LOMA) are shown in black. The last pane shows the syllable labels with font size reflecting the estimated prominence.

The individual signals were *z*-scored and the combinations were obtained as weighted sums of appropriate signals using the following feature weights 1.0, 1.0, and 0.5, for F0, en, and dur, as in Kallio et al. (2018, 2020). The resulting combined signals were subjected to the CWT using a Mexican Hat mother wavelet with scales a quarter of an octave apart. Lines of maximum amplitude (LOMA) were determined for each syllable from 10 scales centered on the average syllable length of the stimuli, yielding the final syllable-based prominence estimates (see Figure 1).

The prominence estimates will be referred to by the feature combinations used for their calculation: F0, dur, en, F0-dur, F0-en, en-dur, and F0-en-dur.

### 2.6 Prominence-based measures

The procedure described in the previous section yields a single (feature combination dependent) numeric prominence value for every syllable in each analyzed utterance. Several measures were tested in this work in order to compare realizations of two utterances of the same sentence.

**Figure 2.**
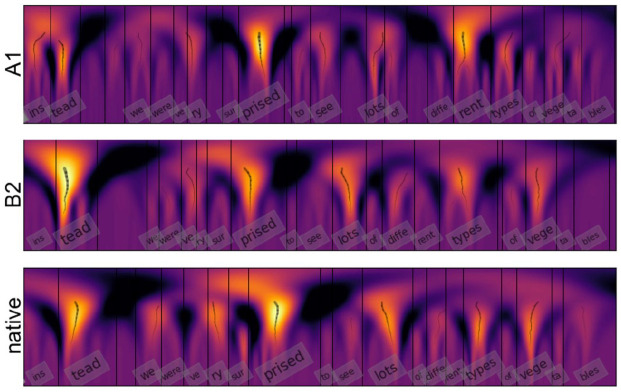
Example of a comparison of syllable prominence produced by a native speaker and two second or foreign language speakers with different proficiency levels (B2 and A1). Syllable prominence is estimated with continuous wavelet transform using a combination of F0, en, and dur. Font size represents the estimated prominence values utilized in correlation measurements.

Figure 2 illustrates the motivation for comparing utterances in terms of prominence. Several syllables in the native speech are clearly realized more prominently than the others (e.g., “tead,” “priced”). The more proficient non-native speaker (B2) has uttered the same sentence in a similar fashion, with the prominence more varied and the same syllables “standing out.” In comparison, for the less proficient speaker (A1), the overall prominence variation pattern is more flat and less similar to the native realization.

Two utterances can be compared by calculating a Pearson correlation coefficient using paired syllable-level prominence values. Alternatively, RMS distance (*z*-scored or not) between the prominence vectors can be used as a measure of similarity.

These two comparison methods were tested in the present work to quantify the degree of similarity between the native and non-native renditions of the same sentences (and, subsequently, comparing the similarity degree with assessment grades). Similar to Kallio et al. (2020), we made two versions of the comparisons: firstly, we evaluated the correlation and RMS distances of each L2 sample to the mean and median prominence values of corresponding L1 utterances. Secondly, each L2 utterance was compared with each L1 production of the same utterance, and the highest correlation and lowest RMS distance with a L1 rendition were selected as measures of similarity.

As in our previous work (Kallio et al., 2020), using the “best” correlation with a native speaker yielded the best results in terms of explaining the assessment grade and was therefore used in the analysis below.

### 2.7 Statistical analysis

All statistical analyses were computed with the R program (R Core Team, 2017). The effects of temporal measures and distances between L1 and L2 syllable prominence estimates to proficiency assessments were studied using a proportional odds logistic regression model (POLR) from the R package MASS (Ripley et al., 2013). The POLR is based on cumulative distribution probabilities and fits well with ordinal categorical data (McCullagh, 1980). This model was selected because the CEFR scale used for L2 prosody assessments is a cumulative description of proficiency and the data is thus ordinal and categorical.

We fitted the POLRs with grade (proficiency levels A1–C2 treated as an ordered factor) as a dependent variable and temporal measures or prominence distance measures as independent variables. For temporal measures, a model combining all measures as well as separate models for each measure was fitted. For prominence distance measures, POLRs were fitted separately for each feature combination used for estimating syllable prominence. In order to take into account the possible differences arising from the speakers’ L1s, the statistical models were centered for each speaker’s L1. We compared the models based on the Akaike information criterion (*AIC*) in order to find out the relevant acoustic properties affecting prosodic proficiency assessment.

## 3 Results

### 3.1 Assessments

The consistency between raters was tested with Cronbach’s alpha from the R package psy (Falissard & Falissard, 2009). The value of Cronbach’s alpha (.94) shows high consistency between the ratings given to each sample by the 40 assessors. The high alpha value shows a relative agreement; thus, the absolute proficiency assessments of individual samples may differ while the scoring patterns are highly consistent among assessors.

**Figure 3.**
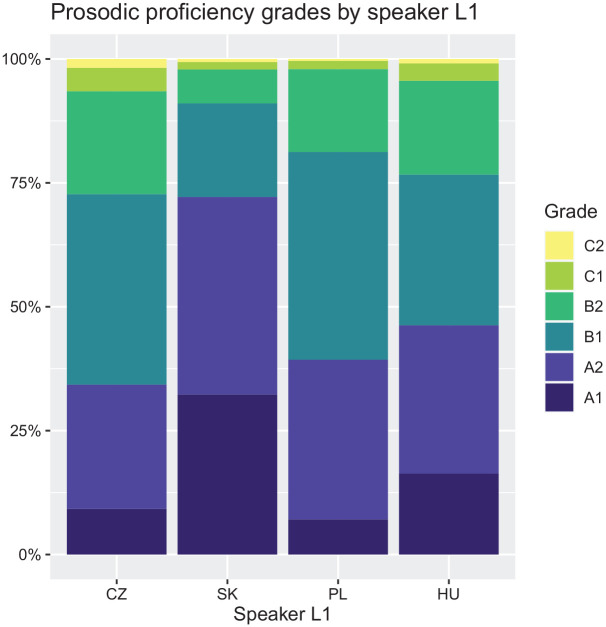
Prosodic proficiency grades by speakers’ native language (L1).

As we did not control for the general language proficiency levels of the participants prior to speech data collection, the prosodic proficiency grade distributions vary among the L1 groups (see Figure 3). The assessments of Slovak samples are skewed towards A-levels, while the majority of Czech and Polish samples were given a B-level assessment. C-level assessments were rare in all L1 groups.

### 3.2 Temporal fluency measures

Collinearity amongst predictors is to be avoided with the proportional logistic regression model. Given the strong relationships among the temporal fluency measures considered here—for example, the frequency and ratio measures for the same type of pauses—we first evaluate the Pearson correlations among the measures. As seen in the correlation table (Table 2), ARTRATE correlates negatively with the pause and correction/repetition measures, and relatively strongly in particular with those for silent pauses (SP-freq and SP-RATIO).

**Table 2.** Correlations of temporal measures.

	ARTRATE	SP-FREQ	SP-RATIO	FP-FREQ	FP-RATIO	CR-FREQ	CR-RATIO
ARTRATE	1	−0.73	−0.67	−0.44	−0.34	−0.48	−0.38
SP-FREQ		1	0.72	0.43	0.2	0.47	0.2
SP-RATIO			1	0.22	0.07	0.28	0.06
FP-FREQ				1	0.83	0.33	0.21
FP-RATIO					1	0.27	0.26
CR-FREQ						1	0.67
CR-RATIO							1

Also, as expected, the correlations between the measures for the same temporal fluency phenomenon are quite high: 0.72 for the silent pause pairs, 0.83 for filled pauses, and 0.67 for correction/repetition. In order to mitigate potential multicollinearity effects and confounding effects mentioned in the introduction, we opted to avoid combining these highly correlated measures in single models. Therefore, here we report the results only involving the *ratio* measures (continuous measures slightly less correlated with ARTRATE), but using the corresponding *freq* measures does not change the results qualitatively.

The relation between the temporal fluency measures and prosodic proficiency assessments was studied by fitting POLRs with grade treated as an ordered categorical dependent variable. The temporal measures ARTRATE, SP-RATIO, FP-RATIO, and CR-ratio plus their interactions with the speaker’s L1 were used as independent variables. To quantify the effect sizes for individual L1s, the fitted models were re-centered for each language separately. Furthermore, the interactions between any given individual measure and speakers’ L1s in the re-centered models were used to compare the effect sizes of the measure on the grade for different languages. In order to estimate the significance levels, *p*-values were further calculated from *t*-values with effective degrees of freedom used by the model (*DF* reported in model-specific summary tables). We chose to use significance levels of *p* = 0.01–0.05* and *p* < 0.01**.

Table 3 summarizes the model fit. The *AIC* value for the fit is 14,865. Given the *AIC* of the corresponding null model, 16,852, the fit explains around 11.8% of variance in the data.

**Table 3.** Summary of the proportional odds logistic regression model with temporal measures as predictors of prosodic proficiency, centered for each speaker’s native language (L1). *DF*: 24. *p*-values: 0.01–0.05*, <0.01**.

L1	ARTRATE		SP-RATIO		FP-RATIO		CR-RATIO	
	Effect	*t*-value	Effect	*t*-value	Effect	*t*-value	Effect	*t*-value
CZ	1.2	12.58**	4.83	5.55**	−11.29	−6.68**	3.67	4.62**
SK	1.75	13.85**	−0.76	−0.73	4.87	1.86*	9.66	5.51**
PL	0.38	3.65**	−4.45	−4.7**	−11.61	−5.79**	−1.06	−0.93
HU	1.53	13.68**	6.64	5.19**	−21.41	−4.94**	2.83	1.73*

The table reveals the general trends as well as some differences (qualitative and quantitative) between the L1 groups. The significantly positive effect of ARTRATE for all L1 groups means that the greater the ARTRATE (the faster the articulation), the better the grade. With the exception of the Polish L1 group, the ARTRATE yields the greatest absolute *t*-values from all independent variables, suggesting that this measure provides the greatest explanatory value in terms of grading. The differences among the reported effect sizes for the different L1 groups are significant, with the exception of the Slovak–Hungarian pair ( t=1.31 ).

For FP-RATIO, the fit demonstrates the expected negative relationship between this measure and the grades: the lower the ratio (the fewer filled pauses) the better grade. The effects for FP-RATIO are all significantly negative, except for Slovak for which the effect size is not significantly different from 0 (absolute *t*-value <2 ). Again, apart from the Czech–Polish pair ( t=0.12) , the differences in effect sizes are all significant. Interestingly, for the Polish group, FP-RATIO provides the strongest predictive power in terms of grading from all considered temporal measures.

The situation is less clear for the two remaining independent variables, with the only significantly negative effect obtained for SP-RATIO for the Polish group. The effect of this measure on grading is significantly positive for the Czechs and Hungarians (non-significant for the Slovaks). Similarly, the fit yields a significantly positive effect for CR-RATIO for the Czechs and Slovaks. Apart from Czech–Hungarian for both SP-RATIO and CR-RATIO ( t=1.17 and 0.46 , respectively) and for Polish–Hungarian for CR-RATIO ( t=1.95 ), the reported effect size differences are all significant. This somewhat counter-intuitive result can likely be attributed to the relatively high correlation between these variables and the other independent measures, ARTRATE in particular.

Also, the occurrence of the disfluencies quantified by the SP-RATIO, FP-RATIO, and CR-RATIO measures varies among the L1 groups: filled pauses are rare (mean FP-RATIO = 0.01) but relatively equally distributed among all L1 groups, while silent pauses are more common in the Slovak L1 group (mean SP-RATIO = 0.17) than in other groups (mean SP-RATIO = 0.12 for all other L1 groups).

Table 4 summarizes the POLRs fitted separately for each individual temporal fluency measure as an independent variable (interacting with speaker L1). These separate fits indeed reveal that on their own, the temporal fluency measures capture the expected effects on the grading: the effect of ARTRATE is significantly positive for all language groups, while the effects of the silent and filled pauses as well as corrections/repetitions are significantly negative (except for FP-RATIO for the Czechs). The reported *AIC* values reveal a considerably better fit for ARTRATE (corresponding to 10.5% variance explained) compared to the remaining measures (between 6.5% and 6.9%).

**Table 4.** Summary of the proportional odds logistic regression models with articulation rate and each temporal measure used separately as a predictor of grade, centered for each speaker’s native language. The *AIC* measure depicts the quality of fit for each model. *DF*: 12. *p*-values: 0.01–0.05*, <0.01**.

	ARTRATE		SP-RATIO		FP-RATIO		CR-RATIO	
	Effect	*t*-value	Effect	*t*-value	Effect	*t*-value	Effect	*t*-value
CZ	0.89	13.54**	−0.08	−2.69**	−0.06	−0.09	−0.98	−12.06**
SK	1.51	25.34**	−0.37	−17.99**	−12.86	−19.83**	−0.84	−12.63**
PL	0.83	11.44**	−0.19	−8.82**	−6.71	−9.46**	−0.63	−9.79**
HU	1.2	15.43**	−0.16	−5.53**	−6.51	−7.12**	−1.23	−9.18**
*AIC*	15,082		15,734		15,694		15,761	

Also, when fitted separately, ARTRATE provides the highest *t*-value for the effect sizes for all L1 groups including Polish, suggesting the greatest explanatory power for this measure in terms of grading.

**Figure 4.**
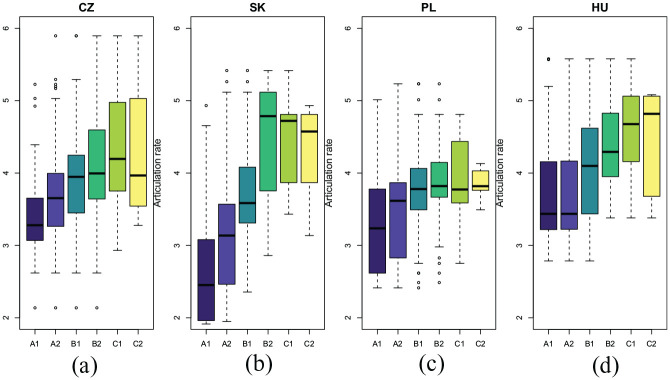
The articulation rates (syllables per second) and prosodic proficiency grades for each speaker’s native language group: (a) Czech; (b) Slovak; (c) Polish; (d) Hungarian.

Figure 4 illustrates the relationship between ARTRATE and assessment grades for individual L1 groups (including the relatively poorest fit for the Poles). For the Czechs, Slovaks, and Poles, ARTRATE seems to “separate” the lower grades better than those for more proficient speakers (B2–C2 range). Interestingly, for Hungarians the situation is somewhat reversed, with essentially identical ARTRATE distributions for A-grades and more pronounced differences for the B1–C2 grade range.

### 3.3 Prominence-based measures

In order to investigate the effect of syllable-level prominence production on prosodic proficiency assessments, we fitted POLRs separately for each feature combination used for CWT-based prominence estimates. The correlation between the CWT-based prominence estimates for L2 speakers with the best native match with interaction with the speaker L1 were used as independent variables; as before, the assessment grade is the dependent variable.

Table 5 summarizes the fits. The effects are generally significantly positive (all negative effects are non-significant with absolute *t*-values less than 2).

**Table 5.** Summary of the proportional odds logistic regression models with each prominence estimation method as a predictor of prosodic proficiency, centered for each speaker’s native language (L1). The *AIC* measure depicts the quality of fit for each model: *AIC* for the null model was 16,852. *DF*: 12. *p*-values: 0.01–0.05*, <0.01**.

L1	F0		en		dur		F0-en		F0-dur		en-dur		F0-en-dur	
	Effect	*t*-value	Effect	*t*-value	Effect	*t*-value	Effect	*t*-value	Effect	*t*-value	Effect	*t*-value	Effect	*t*-value
CZ	1.26	6.51**	0.33	1.24	5.87	13.81**	1.74	7.34**	1.15	5.42**	0.57	1.76	1.54	5.9**
SK	2.04	7.63**	1.77	6.92**	3.99	11.97**	1.68	7.61**	2.73	9.27**	2.71	8.88**	2.12	7.75**
PL	1.45	5.81**	−0.39	−1.5	0.64	2.22*	0.16	0.61	1.82	5.73**	−0.19	−0.69	−0.16	−0.54
HU	1.56	6.03**	2.38	7.56**	1.48	3.56**	2.03	7.38**	1.92	6.19**	2.44	7.56**	2.4	7.9**
*AIC*	16,086		16,146		15,909		16,090		16,070		16,113		16,098	

Comparing effect sizes for different fits (using the interaction with L1 as above) reveals an interesting pattern. For F0-based estimates, the effect sizes are not significantly different from each other, except for the Czech–Slovak pair ( t=2.36,p<0.05 ). For dur, the effect sizes are *all* significantly different among the L1 groups except for the Polish–Hungarian pair ( t=1.65,p>0.05 ) with lower effect sizes than the other two languages. The effect sizes for en and en-dur divide the L1 groups into two “sets”: the Slovak–Hungarian and Czech–Polish pairs, with non-significant differences within each pair and significantly greater effects for the former group compared to the latter one. Finally, for the remaining feature combinations, F0-en and F0-en-dur, the effect sizes are not significantly different within the Czech–Slovak–Hungarian set (except for the Czech–Hungarian pair for F0-en-dur with t=2.15,p<0.05 ) but are significantly lower for the Polish L1 group.

For the Czech and Slovak L1 groups, the model with dur estimate as a predictor yields the highest *t*-value among the predictors; for Polish the highest *t*-value was provided by the F0-based fit, and for Hungarian by the F0-en-dur.

**Figure 5.**
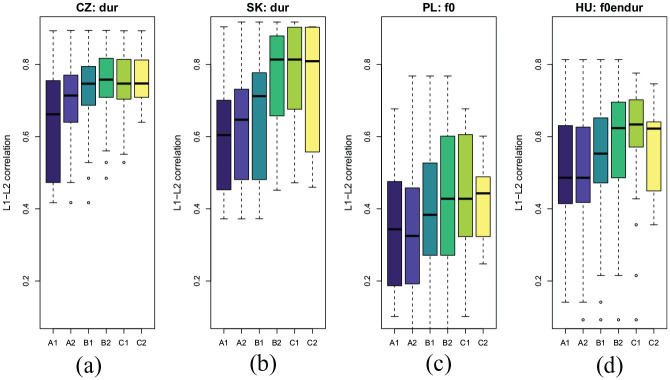
The distributions of correlations to native prominence estimates with the best native language (L1)-specific feature combination and prosodic proficiency grades for each speaker’s L1 group: (a) Czech; (b) Slovak; (c) Polish; (d) Hungarian. L2: second or foreign language.

Figure 5 depicts the relationship between the assessment grades and the prominence correlations for the “best” feature combination for individual language groups.

### 3.4 The contribution of prominence-based measures

The *AIC* values for the models reported in Table 5 reveal the considerably worse quality of fit for the prominence-based measures compared to the ARTRATE and temporal fluency based measures. Given the *AIC* of the null model (16,852), these fits explain between 4.19% (en) and 5.60% of variance.

This observation gives rise to the question of whether the prominence-based measures add any explanatory power in terms of assessment over the more traditional temporal measures.

In order to address this question, we fitted POLRs combining the temporal fluency measures and prominence-based measures (one feature combination per model) as independent variables (with interactions with the L1 group) and used log-likelihood comparisons to compare them with the models using the temporal fluency measures only. All models with additional prominence-based measures provided significantly better fits regardless of the feature combination used ( p<0.001 ).

Comparing the *AIC* measures of these models shows a quantitatively relatively meagre benefit from including prominence-based measures in terms of explained variance. While the *AIC* for the model with only temporal fluency measures (Table 4) corresponded to 11.8% of explained variance, including the prominence-based features contributed to an increase from 11.9% (for F0-EN and F0-EN-DUR) to 12.4% for the dur signal.

## 4 Discussion

This study investigated how syllable-level prominence realizations predict prosodic proficiency of L2 speakers with different L1 backgrounds compared to more widely used temporal fluency measures. The results showed that temporal measures, especially articulation rate, have stronger predictive power than syllable-level prominence realizations (RQ1 and RQ2). However, statistical models with temporal measures augmented with prominence-based measures explained the assessment results significantly better than the models with only the temporal measures. This indicates that syllable-level prominence realizations bring complementary information to the evaluation of L2 prosody (RQ3).

Despite the variation in the research on temporal fluency in L2, most studies have found speed measures such as speech or articulation rate to be amongst the best predictors of fluency or prosodic proficiency (Cucchiarini et al., 2002; de Wet et al., 2009; Kallio et al., 2017; Kang, 2010; Lennon, 1990). Our results are in line with these findings, supporting the importance of articulation rate as a predictor of prosodic proficiency. Using the articulation rate together with disfluency–time ratios, however, resulted in quantitatively significant differences in the effects of the measures among L1 groups (including opposite effects for disfluency–time ratios, as shown in Table 3). However, the results for the individual measures modeled separately match the expectations of articulation rate correlating positively and the remaining measures correlating negatively with proficiency level (Table 4). The results of the model with all temporal measures (summarized in Table 3) could be attributed to the relatively high correlation between articulation rate and the disfluency measures. Although articulation rate and, for example, SP-RATIO, are not directly related measures, it is possible that speakers who articulate faster pause less, and vice versa.

The significance of individual temporal measures also varied among L1 groups when modeled separately. This was expected and can, on one hand, be partly attributed to the varying use of silent pauses (which were more common in the Slovak L1 group than in other L1 groups), and on the other hand to the rare occurrence of other disfluencies in the analyzed speech samples. When filled pauses, corrections, and repetitions do occur in speech, they might have a significant effect on prosodic proficiency assessments. However, since the ratio values remained very low (mean FP-RATIO = 0.01 for all L1 groups, mean CR-ratio = 0.05 for the Czech, 0.03 for the Slovak and Polish, and 0.02 for the Hungarian L1 groups), the predictive power of these measures should be examined critically. Our measures also remain insensitive to differences within disfluency types, such as the placement or length of an individual pause. These aspects, which could be important in the perception of fluency, should be investigated further with larger data and longer speech samples: in this study, the size of the speech data allowed the investigation of only the overall occurrence of disfluencies.

It is important to note that the data analyzed in this study consisted of read speech. Read speech was selected because it allows the comparison of the prominence realizations of same syllables in the same context between different speakers. However, read speech differs from spontaneous speech with regards to temporal features: on one hand, the articulation rate can be faster in spontaneous than read speech (Cucchiarini et al., 2010). On the other hand, pauses are more frequent in spontaneous than read speech, which can reduce the importance of articulation rate as an indicator of fluency (Cucchiarini et al., 2002). Perhaps due to the controlled nature of read speech, disfluency-related measures are more reliable in predicting fluency and prosodic proficiency in read speech than in spontaneous speech (Cucchiarini et al., 2002; Kallio et al., 2017).

The results for the prominence measures reveal interesting similarities and differences among the L1 groups (see Table 5). The best feature combinations for predicting L2 proficiency in the current study differed depending on the speaker’s L1, but they also differed from the best feature combination in Kallio et al. (2020). These results complement our previous findings in Kallio et al. (2020) and encourage taking into account the effect of the speaker’s L1 on the production of L2 prominence when studying L2 prosody and developing teaching and assessment methods. Based on our previous results (Kallio et al., 2020) and the stress characteristics of the target language, English, we expected duration to be involved in the best feature combinations for capturing syllable prominence characteristics relevant for assessing L2 proficiency. This was the case for the Czech, Slovak, and Hungarian L1 groups, but not for the Polish speakers. Czech, Slovak, and Hungarian have a phonemic quantity distinction, which may hinder the use of duration as a cue for signaling prominence in the respective languages (Remijsen, 2002) as well as in L2 English.

The two most closely related L1s, Czech and Slovak, shared duration as the strongest predictive prominence feature, although the effect sizes differed significantly. Our analysis did not reveal whether the different effect sizes can be attributed to language-specific tendencies or to the different proficiency distributions of the two L1 groups. However, our result supports the findings of Bilá and Zimmermann (1999) and Weingartová et al. (2014): proficiency levels could be predicted from how the speakers of these languages use duration to differentiate stressed and unstressed syllables in English.

For the Hungarian L1 group, interestingly, the best combination was with all three prosodic signals (F0-EN-DUR). The Hungarian speakers have consistently high effect sizes for all feature combinations involving energy, while duration alone yielded the lowest (yet significant) *t*-value. Because Hungarian, like Czech and Slovak, has a phonemic distinction of quantity (Hungarian for both vowels and consonants) and researchers have questioned the role of duration in signaling prominence in Hungarian (Vogel et al., 2015), we expected to find a stronger effect for the duration signal in predicting prosodic proficiency in L2 English. However, Hungarian differs from the other L1s in this study with regards to sentence stress: although English sentence stress varies generally in more than the ones of the L1s involved in this study, all the languages except Hungarian have the tendency to place the most prominent part of a phrase at the final position (Dubeda & Mády, 2010; Eschenberg, n.d.; Roach, 2000). Hungarian F0 contour patterns also have a relatively sharp falling tendency, as opposed to the other L1s (Benuš et al., 2014; Dubeda & Mády, 2010; Eschenberg, n.d.; Varga, 2002). Since prominence patterns also arise from the sentence level, the differences in phrasal tendencies may affect the acoustic realizations of stress at the syllable level.

For the Polish speakers, the best predictive feature was F0, which has previously been found to be the most relevant cue in marking prominence in Polish (Jassem, 1962; Malisz & Wagner, 2012). It is possible that Polish speakers transfer the tendency to use F0 as the primary cue for prominence from their L1 to English, making it a potential feature in capturing the raters’ distinctions between less proficient and advanced speakers. It should be noted, however, that the effect sizes were in general lower for Polish than for the other L1 groups, regardless of the model. The implications based on syllable prominence estimates should thus be treated with caution, and further investigation on the matter is recommended. The low effect sizes could indicate variation in the acoustic prominence realizations within this group, or they may stem from weak manifestation of Polish word and sentence stress (Cwiek & Wagner, 2018; Dogil & Williams, 1999).

For the feature combinations F0-EN and F0-EN-DUR, the effect sizes were relatively similar among the Czech, Slovak, and Hungarian L1 groups. This may stem from the prosodic similarities among these languages, indicating that the CWT method could reveal interesting information about the similarities and differences among languages realized at the syllable level. It is also encouraging that the most closely related languages here—Czech and Slovak—behave in a relatively similar manner in terms of the effects of the CWT-based measures on proficiency assessments. The fact that the Polish L1 group stands out as different from the other L1 groups may stem from language-specific characteristics our analyses were unable to reveal, or from variation within the Polish L1 group.

It should be noted that the native English speakers, used as a reference group in this study, consisted of four British English speakers and two American English speakers. We acknowledge that the number of speakers should be larger in order to give reliable representations of prominence realizations in the two varieties. However, by comparing each L2 utterance to all native English productions of the same utterance and choosing the native rendition with the highest correlation to the L2 production, we avoid possible bias that could emerge from mean or median prominence values of all native speakers.

In our data, the higher proficiency levels (C1–C2) were rare in all L1 groups, which influenced statistical analyses for the higher grades. In general, the selected methods were able to distinguish less proficient (A-level) and advanced (C-level) speakers. However, distinguishing between neighboring levels, especially at the higher end of the scale, proved more challenging. This was also the case in our previous studies using the same CEFR scale (Kallio et al., 2017, 2018, 2020). We suspect one reason for this to be the cumulative nature of the rating scale: the differences between proficiency levels are greater at the lower part of the scale because the language learning process is seen as progressive or accumulative. A saturation effect is also possible, as it is generally more challenging even for human raters to consistently differentiate between the levels of highly proficient speakers. We also want to note the difference in the occurrence of B-level assessments among the L1 groups: Slovak speakers got B-level assessments about 50% less often than all the other L1 groups, which may have influenced the results. Since the overall proficiency levels of the speakers were not controlled for, our samples do not necessarily represent the general English speaking proficiency of the language groups. However, the frequency of silent pauses within the Slovak L1 group could be attributed to lower proficiency levels in that group.

The consistency among assessors was very high in our study (Cronbach’s alpha = 0.94), despite the fact that we used non-professional and non-native raters. Various factors may have led to this high relative agreement: read speech is easier to assess, since the raters do not have to pay attention to grammar and vocabulary. Our raters were also trained identically and provided with specific instructions and scale for assessments, which have previously been shown to increase inter-rater reliability (Brennan & Brennan, 1981; Huang et al., 2016). The choice of the assessors might, however, have influenced our results. Non-professional raters seem to emphasize fluency features more than professional raters, although all raters find both fluency and accuracy to be important criteria for spoken L2 proficiency (Duijm et al., 2018). Regardless of expertise, however, raters may emphasize different features in speech, consciously or unconsciously (Kallio et al., 2017; Kormos & Dénes, 2004). We also acknowledge that the raters’ L1 might be another source of variance, although Huang et al. (2016) found no significant effect of rater’s L1 background on their ratings of L2 speakers’ overall proficiency. Investigating the impact of assessor L1 to proficiency ratings was beyond the scope of this article; however, we plan to address this issue in the future.

The results concerning the predictive power of CWT-based prominence estimates in this study differ from our previous research, where syllable prominence served as a more reliable predictor of prosodic proficiency (Kallio et al., 2020). Possible reasons for this can be looked for in the differences between research data. Firstly, the target utterances in Kallio et al. (2020) were prosodically more suited for studying stress production, as they included several minimal pairs differing only in word stress. This aspect was not considered when recording speech data for the current study, where more emphasis was given to the natural flow of the read story. Secondly, the target language in the previous study (Finland Swedish) differs prosodically from the target language in the current study (English). While both languages have relatively strong stress realizations with vowel reduction in unstressed syllables (Bolinger, 1965; Fant & Kruckenberg, 1994), the importance of stress and a complex syllable structure makes English stand out (Dauer, 1983; Ladefoged & Johnson, 2014). Also, Finland Swedish shares many prosodic characteristics with Finnish (unlike Sweden Swedish), meaning that the prominence-based measures may have captured more subtle differences in the Finnish speakers’ proficiency. When estimating syllable prominence in L2 English, features complementing the traditional prosodic measures—such as RMS energy range and F0 slope (Tepperman & Narayanan, 2005), spectral tilt (Ferrer et al., 2015), or relative sonority levels (Yarra et al., 2017)—could improve the results.

The contribution of the local prominence-based measurements was relatively small compared to the more traditional temporal features. Nevertheless, our findings (alongside the earlier ones by Kallio et al., 2020) show the potential of prominence estimation and the underlying CWT-based method for the assessment of prosodic proficiency. However, the CWT-based method seems to work better with controlled data specifically designed for studying stress production. Although the temporal measures served as better predictors of prosodic proficiency in our data, syllable-level prominence measures provided L1-specific information that is potentially useful for the assessment of L2 speaker proficiency. Therefore, we recommend that the acoustic features underlying non-native stress production should be scrutinized in more detail. Cross-linguistic studies on L2 speech with relating L1s have been marginal so far, but the findings of the current study suggest that prosodic differences can be found in the L2 speech of the speakers even from typologically close languages. Moreover, our results support the importance of the speaker’s L1 for designing robust and reliable assessment methods for L2 speech, as well as for the customization of teaching and learning tools.

## 5 Conclusions

This study compared traditionally used temporal measures and a relatively new method based on syllable prominence estimates as predictors of prosodic proficiency in non-native speakers of English with respect to their L1 backgrounds. Our results show that temporal measures, articulation rate in particular, serve as good predictors of prosodic proficiency of L2, while local prominence-based measures contribute to the quality of proficiency prediction. The predictive power of the measures varies with regards to speaker’s L1, especially for the syllable-level prominence. This indicates that L2 learners use different acoustic cues in prominence production depending on their L1. This issue remains little addressed in the existing research literature, as stress remains underrepresented in many L2 assessment systems and criteria. The present study reports on a preliminary attempt to untangle the acoustic complexity of L1 transfer to L2 stress production. We conclude that the effect of speaker’s L1 on the production of temporal features as well as syllable prominence in L2 should be taken into account when studying L2 prosody and developing teaching and assessment methods.
